# Impact of the perioperative inotropic support in grown-up congenital heart patients undergoing cardiac surgery: a propensity score adjusted analysis

**DOI:** 10.1186/s13613-020-00709-0

**Published:** 2020-07-09

**Authors:** Philippe Mauriat, Mirela Bojan, Sylvie Soulie, Hélène Foulgoc, Nadir Tafer, Alexandre Ouattara

**Affiliations:** 1 Department of Anaesthesia and Critical Care, University of Bordeaux, Haut-Levêque Hospital, Avenue Magellan, 33000 Pessac, France; 2 Department of Anaesthesia, Congenital Cardiac Unit, Marie-Lannelongue Hospital, Paris-Sud University, 133 Avenue de la Résistance, 92350 Le Plessis-Robinson, France; 3 Department of Anesthesia and Critical Care, Louis Pradel Hospital, University of Lyon, 59 Boulevard Pinel, 69500 Bron, France; 4 Biology of Cardiovascular Diseases, University of Bordeaux, INSERM, UMR 1034, 33600 Pessac, France

**Keywords:** Grown-up congenital heart, Postoperative low cardiac output syndrome, Inotropic-vasoactive support, Levosimendan, Milrinone, Adult congenital cardiac surgery, Mechanical ventilation, Intensive care stay

## Abstract

**Background:**

Grown-up congenital heart (GUCH) patients represent a growing population with a high morbidity risk when undergoing reparative surgery. A main preoperative feature is right ventricular failure, which represents a risk factor for postoperative low cardiac output syndrome. Levosimendan has a potentially beneficial effect. This retrospective study included consecutive GUCH patients with surgeries in a tertiary cardiothoracic centre between 01-01-2013 and 01-10-2017, to test the hypothesis that the postoperative use of levosimendan might be associated with shorter time of mechanical ventilation, when compared with the use of milrinone. To adjust for bias related to the probability of treatment assignment, it uses the inverse propensity score weighting methodology.

**Results:**

Overall 363 patients had GUCH surgeries during the study period, their mean age was 31.39 ± 15.31 years, 87 patients were eligible for analysis in the Levosimendan group and 117 in the Milrinone group. The propensity score used pre- and intraoperative variables and resulted in a good balance between covariates. The Levosimendan group included patients with higher preoperative risk scores, a higher prevalence of left and right ventricular failure, who required more often the addition of epinephrine, renal replacement therapy, prolonged mechanical ventilation and intensive care stay. However, after propensity score weighting, patients in the Levosimendan group had shorter durations of mechanical ventilation (average treatment effect − 37.59 h IQR [− 138.85 to − 19.13], p = 0.01) and intensive care stay (average treatment effect − 3.11 days IQR [− 10.03 to − 1.48], p = 0.009). The number of days of additional epinephrine support was shorter and the vasoactive inotropic scores lower.

**Conclusion:**

We report a beneficial effect in terms of duration of mechanical ventilation and intensive care stay, and on inotropic requirements of the use of levosimendan following GUCH surgeries. The use of levosimendan in this setting requires validation at a larger scale.

## Introduction

Grown-up congenital heart (GUCH) patients represent a growing population with complex pathophysiology due to decades of living with abnormal cardiac anatomy, which result in comorbidities affecting all organ systems. Therefore, they are at higher risk of postoperative complications when compared with patients with acquired cardiac diseases [1–4]. When undergoing surgery in early infancy, about 25% of congenital heart patients experience postoperative low cardiac output syndrome (LCOS) [5]. Management of postoperative LCOS includes inotropic-vasoactive support [6], and sometimes mechanical support. Together with beta-agonists and phosphodiesterase-inhibitors, levosimendan has been used in adults and children with LCOS following cardiac surgery. It has been found beneficial in adults with severely reduced left ventricular (LV) systolic function [7], and safe and beneficial in children with congenital cardiac operations [8–10]. The pathophysiology of heart failure in GUCH patients is different from that of acquired heart diseases, and they have a high prevalence of right ventricular failure [11]. Therefore, the pulmonary vasodilatatory effect of levosimendan, as well as the positive inotropic and lusitropic effects is particularly appealing in the context of GUCH surgeries. Based on these considerations, we conducted a retrospective study to test the hypothesis that GUCH patients treated by levosimendan for LCOS following cardiopulmonary bypass (CPB), compared to those having received a conventional inotropic-vasoactive support based on milrinone, might be associated with shorter time of mechanical ventilation.

## Materials and methods

This retrospective analysis was conducted in a cohort of consecutive GUCH patients older than 15 years, undergoing surgery between January 1st 2013 and October 3rd 2017 at the University Hospital of Bordeaux, France. The Ethical Committee of the French Anaesthetic Society waived the need for written informed consent of the patients to perform this retrospective analysis, after de-identification of all patient data (reference IRB 00010254 - 2019 - 059). Only patients undergoing surgery with CPB and aortic cross-clamping, and with complete postoperative records were analysed.

Because of the absence of a GUCH-specific risk score, the baseline risk category was assessed by the Euroscore II. Its use has, nevertheless, been shown to result in an underestimation of the perioperative risk [12]. Anaesthesia was induced and maintained using a target controlled infusion of propofol and remifentanil, and with cisatracurium. The arterial and central venous pressure, as well as the venous oxygen saturation and cerebral oxygen saturation were monitored as part of the institutional protocol. Transesophageal ultrasonographic assessment was used in all patients. Normothermic non-pulsatile cardiopulmonary bypass (CPB) was performed using a roller pump and full heparinization (300 U kg^−1^ heparin and an ACT requirement > 400), the pump flow was set at 3.0–3.5 L min^−1^ m^−2^. Warm blood cardioplegia every 20 min and monitoring of the cardioplegic perfusion was performed in all cases, the reinfusion interval was shortened to 12 min in case of ventricular hypertrophy. Monitoring of the pulmonary arterial pressure and of the left atrial pressure was decided at the end of CPB by the attending anaesthesiologist and surgeon according to the underlying pathology and to the preoperative myocardial function.

At the end of CPB, the inotropic-vasoactive strategy was decided by the attending anaesthesiologist, according to the underlying pathology, the preoperative myocardial function, the duration of CPB and cross-clamping and the hemodynamic and ultrasonographic assessment. Patients received either no inotrope, milrinone 0.5 to 1 mcg kg^−1^ min^−1^ or levosimendan 0.2 mcg kg^−1^ min^−1^ during 24 h, and additional low dose (0.02 to 0.05 mcg kg^−1^ min^−1^) epinephrine or norepinephrine, if required. The patients who have not received any inotropic-vasoactive support were not analysed here. Accordingly, the patients were analysed in the Milrinone or the Levosimendan group. Postoperatively, the inotropic-vasoactive support was discontinued according to the daily hemodynamic, ultrasonographic and biological assessment. The vasoactive and inotropic score (VIS) was calculated for the first 4 postoperative days. The Clinical Pulmonary Infection Score (CPIS) [13] was used to diagnose ventilator-associated pneumonia. Renal replacement therapy was available for patients with severe postoperative kidney injury. When required, mechanical ventricular assistance was provided using intra-aortic balloon pump therapy (IABP) or extracorporeal membrane oxygenation (ECMO).

### Outcomes

The primary outcome was the duration of mechanical ventilation. This endpoint was chosen because only patients haemodynamically stable without significant organ dysfunction are extubated. Secondary outcomes included epinephrine requirement, VIS, CPIS within 48 h, requirement for renal replacement therapy, duration of ICU and hospital stay.

### Statistical analysis

All baseline, intra- and postoperative data were available in the institutional database, in which was stored daily information provided by the attending physicians. Continuous normally distributed variables were expressed as means and standard deviations (SD), otherwise as medians and inter-quartile ranges (IQR) and compared with the Student’s t or Mann–Whitney U tests, as appropriate. Categorical variables were expressed as numbers and percentages and compared with the Chi-square test or Fisher’s exact test, as appropriate. Analysis was conducted in intention to treat.

To avoid bias due to the huge differences observed between characteristics of the control patients and the others, the analysis was restricted to the comparison of the Levosimendan and Milrinone groups. The inverse probability of treatment weighting (IPTW) method was used to control for bias due to selection of patients placed on levosimendan or on milrinone. First, a propensity score model included pre- and intraoperative characteristics which could have influenced the decision of starting levosimendan rather than milrinone at the end of CPB. Then, the contribution of each subject was weighted by 1/propensity score in the Milrinone group, and by 1/(1-propensity score) in the Levosimendan group. These weights assured that, for each combination of the covariates used in the propensity score model, the sum of the contributions of all subjects is equal. Balance on covariates between groups after the IPTW weighting was assessed by computing their standardized differences, and groups were considered balanced if the standardized differences were < 0.25. Short-term outcome variables were compared using IPTW-weighted regression models. Results were expressed as absolute average treatment effect, and 95% confidence intervals were estimated by bootstrapping with 500 re-samples. Statistical significance was set at p < 0.05. Analyses were performed using the basic R software package and the “survey” package (https://www.r-project.org).

## Results

**Fig. 1:**
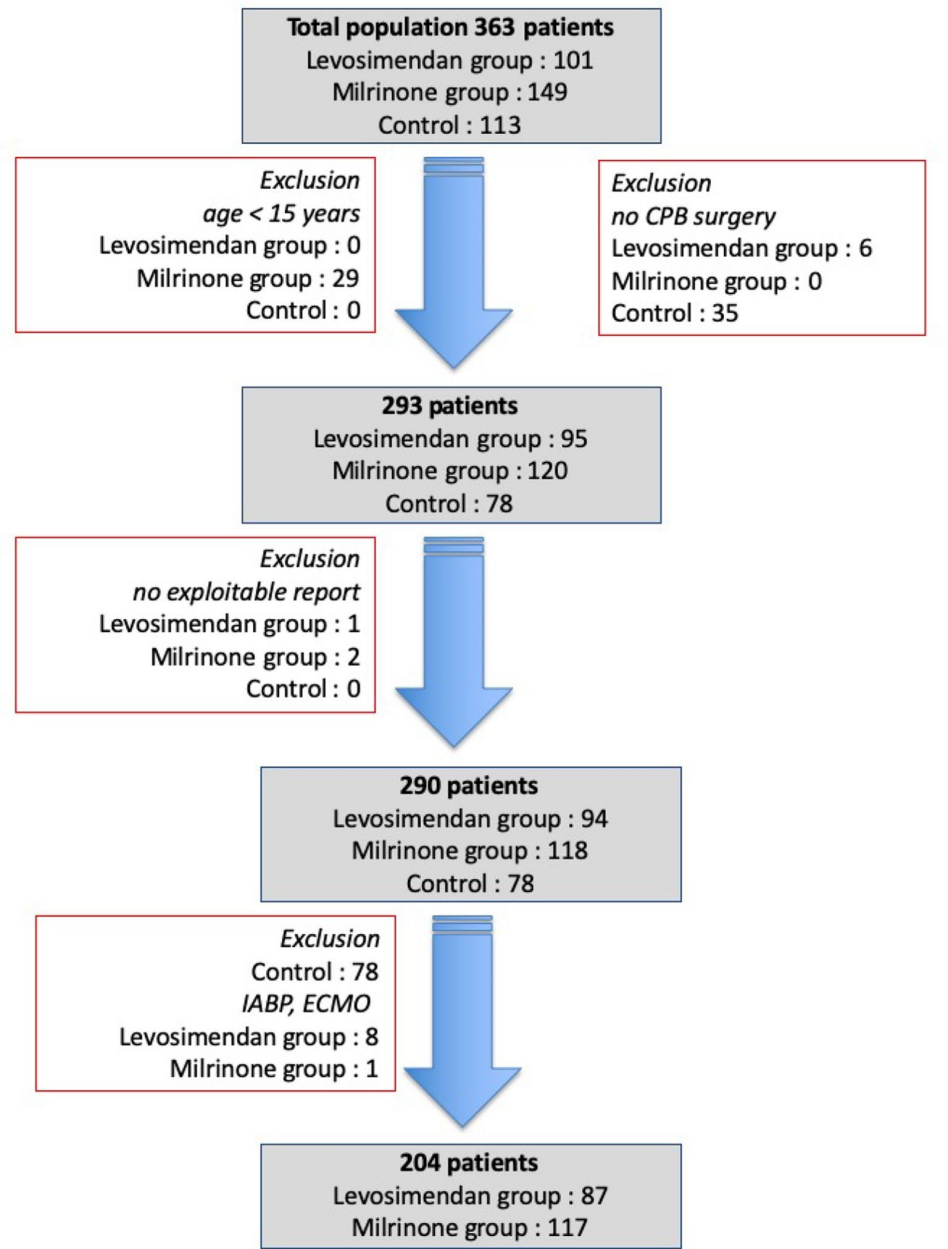
Flowchart of the study cohort

**Fig. 2:**
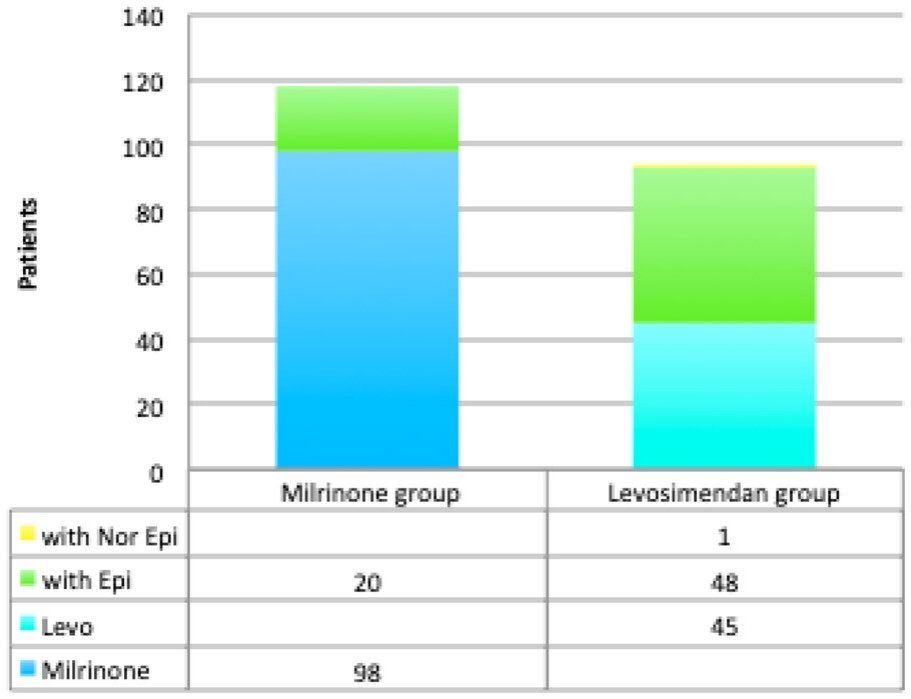
Distribution of the inotropic-vasoactive support among the Milrinone and the Levosimendan groups. Overall, 49.3% of the patients received one inotropic-vasoactive agent, and 23.8% received two agents

Overall, 363 GUCH surgeries were performed during the study period. The patients were on average 31.31 ± 15.63 years old. No patient died within 30 days of admission. As shown in Fig. 1, only 204 patients were analysed here: 117 in the Milrinone group and 87 in the Levosimendan group. As shown in Fig. 2, 49.3% of all patients received one inotropic-vasoactive agent, and 23.8% received two agents. In one patient started on milrinone and epinephrine, levosimendan was introduced on day 2. Overall, 7 patients were placed on IABP (6) or ECMO (1) at the end of CPB, concomitant with starting the inotropic-vasoactive support: 6 in the Levosimendan group and 1 on the Milrinone group. To avoid bias related to the association between this outcome and the postoperative use of inotropes, these patients were not further analysed. Another 2 patients in the Levosimendan group required a second surgery at postoperative day 2 and were placed on ECMO at the end of their second CPB, they were not analysed either, leaving under analysis 87 patients in the Levosimendan group and 117 patients in the Milrinone group. No other patient required mechanical ventricular assistance.

**Fig. 3:**
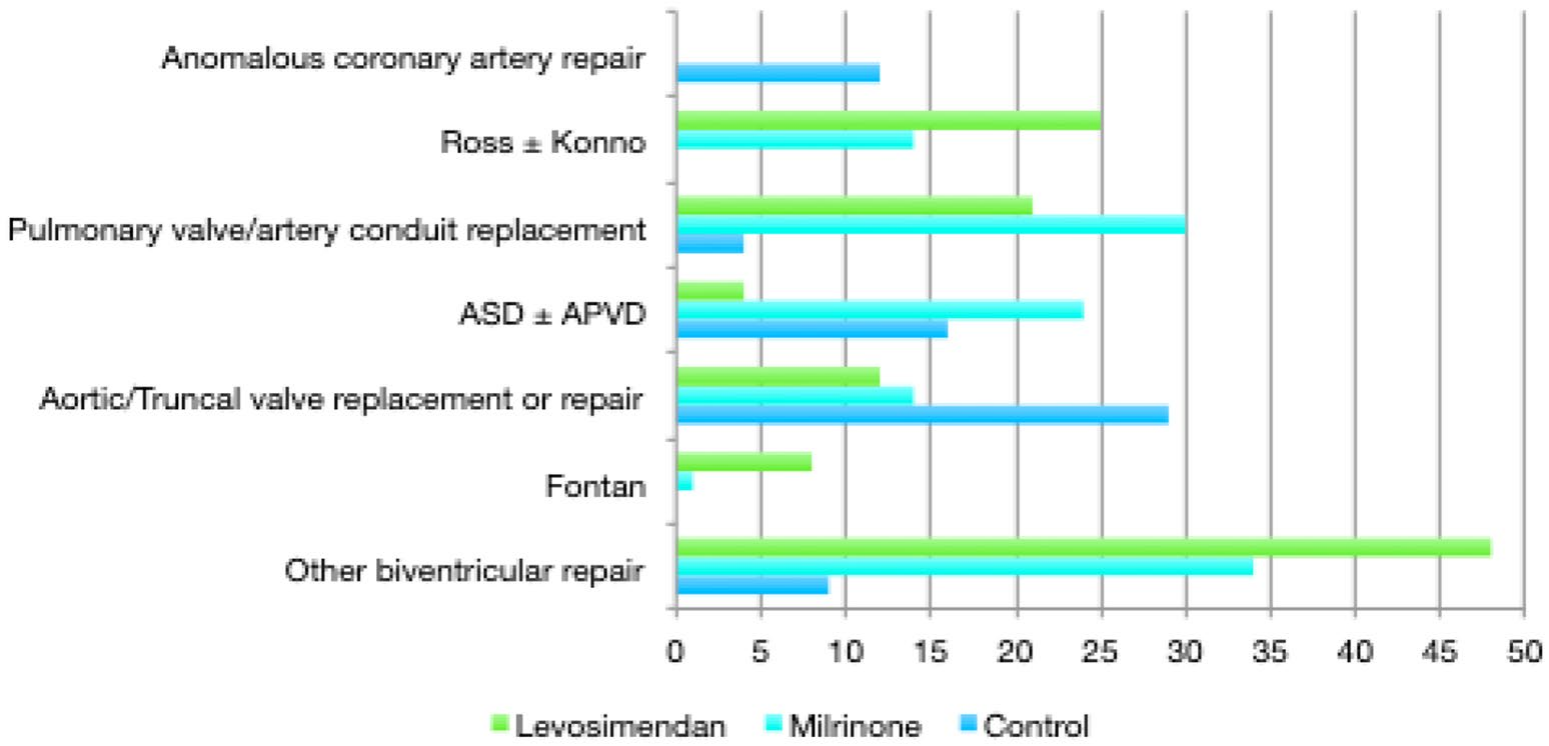
Distribution of the inotropic-vasoactive support according to the underlying pathology and procedure. *ASD* atrial septal defect, VSD: ventricular septal defect

The baseline, intra- and post-operative characteristics of the study population are shown in Table 1. The underlying pathologies and surgical interventions are shown in Fig. 3, together with the distribution of the inotropic-vasoactive support. The variables used in the propensity score model are shown in Table 2, together with their standardized differences before and after IPTW. The propensity score model was well calibrated (Hosmer–Lemeshow test *P* value = 0.59) and had good discrimination (C-index = 0.809). As shown in Table 1, patients in both groups had a high incidence of RV anomaly (failing, hypoplastic, hypertrophic or dilated): 89% in the Milrinone group and 74% in the Levosimendan group. Patients in the Levosimendan group had the highest preoperative risk scores, the highest rates of left and/or right ventricular dysfunction, and underwent redo-surgery more often. As shown in Table 1, they had the longest CPB and cross-clamping durations too. After IPTW weighting, there was a good balance between the pre- and intraoperative variables (Table 2).

**Table 1:** Baseline, intraoperative and postoperative variables among the treatment groups

Variables	Levosimendan (*n* = 94)	Milrinone (*n* = 118)	*P* value
Baseline			
Age (years)	27.49 ± 15.00	32.76 ± 14.89	*0.01*
Weight (kg)	60.21 ± 15.26	66.93 ± 17.34	*0.003*
Redo-surgery	60 (0.64)	61 (0.52)	0.09
Euroscore II	4.76 ± 8 76	2.13 ± 1 73	*0.005*
NYHA	2.66 ± 0.78	2.3 ± 0.78	*< 0.001*
LV ejection fraction (%)	60.47 ± 14.71	64.58 ± 8.66	*0.02*
LV hypertrophy	28 (0.30)	13 (0.11)	*< 0.001*
LV dilatation	9 (0.10)	15 (0.13)	0.52
RV function altered	22 (0.23)	26 (0.22)	*0.007*
RV hypoplastic	7 (0.07)	0	0.29
RV hypertrophy	5 (0.05)	12 (0.10)	*0.05*
RV dilatation	36 (0.42)	67 (0.57)	0.81
Pulmonary hypertension	8 (0.09)	9 (0.08)	0.06
Year of operation: 2013	11 (0.12)	33 (0.28)	
2014	20 (0.21)	21 (0.18)	
2015	20 (0.23)	16 (0.14)	
2016	27 (0.28)	30 (0.25)	
2017	16 (0.16)	18 (0.15)	
Intraoperative			
CPB duration (min)	187 ± 87	115 ± 51	*< 0.001*
Cross-clamping duration (min)	79 ± 60	54 ± 42	*< 0.001*
Postoperative			
Number of days on epinephrine	1 [0–2.75]	0 [0–1]	*0.03*
VIS on postoperative day 1	10.21 ± 10.52	10.06 ± 11.51	0.92
VIS on postoperative day 2	6.31 ± 8.48	6.25 ± 9.78	0.96
VIS on postoperative day 3	3.9 ± 6.87	3.14 ± 9.1	0.50
VIS on postoperative day 4	2.25 ± 5.38	2.16 ± 9.37	0.93
CPIS within 48 h of admission	1.64 ± 2.19	1.50 ± 1.83	0.70
Duration of mechanical ventilation (h)	12 [5.25–34.50]	4 [2–8]	*< 0.001*
Requiring renal replacement therapy	8 (0.09)	3 (0.03)	0.06
IABP or ECMO	8 (0.09)	1 (0.01)	*0.05*
Duration of intensive care unit stay (days)	4 [3–8]	3 [2–4]	*< 0.001*
Duration of hospital stay (days)	13 [9–21]	10 [8–13]	*< 0.001*

Data are shown as means ± standard deviations, or as numbers and proportions. All *P* values were estimated using the Student’s *t* test, the *χ*^2^ or the Fisher test. Statistically significant results are shown in italics

*CPB* cardiopulmonary bypass, *CPIS* Clinical Pulmonary Infection Score, *ECMO* extracorporeal membrane oxygenation, *IABP* intra-aortic balloon pump therapy, *LV* left ventricle, *NYHA* New York Heart Association, *RV* right ventricle, *VIS* vasoactive inotropic score

**Table 2:** Variables used in the propensity score model: balance before and after inverse probability of treatment weighting

Variable	Before IPTW weighting				After IPTW weighting	
	Levosimendan (*n* = 87)	Milrinone (*n* = 117)	Standardized difference	*P* value*	Standardized difference	*P* value**
Euroscore II	3.81	2.12	0.35	0.02	0.17	0.86
NYHA category	2.59	2.30	0.37	0.009	0.22	0.49
LV ejection fraction	0.61	0.64	− 0.28	0.05	− 0.08	0.59
LV dilatation	0.09	0.12	− 0.09	0.52	− 0.07	0.63
RV function altered	0.39	0.21	0.33	0.02	0.08	0.61
RV hypertroph	0.03	0.10	− 0.27	0.05	− 0.02	0.90
RV dilatation	0.57	0.56	0.02	0.90	0.13	0.47
CPB duration	183.23	114.60	0.97	< 0.001	− 0.05	0.82

*CPB* cardiopulmonary bypass, *IPTW* inverse probability of treatment weighting, *LV* left ventricle, *NYHA* New York Heart Association, *RV* right ventricle

*Estimated using a *t* test or a Mann–Whitney test, as appropriate

**Estimated using a weighted regression model

The analysis of the outcome variables is shown in Table 3. Before weighting (Table 1), patients in the Levosimendan group required more often additional epinephrine and had longer durations of epinephrine infusion, they required more often renal replacement therapy and mechanical ventricular support, and they had longer durations of mechanical ventilation and longer durations of ICU and hospital stay. However, after IPTW weighting (Table 3), there was a significantly shorter duration of mechanical ventilation and ICU stay in the Levosimendan group. After IPTW weighting, patients in the Milrinone group had significantly higher VIS within the first 4 postoperative days. Although not statistically significant, there was a shorter duration of additional epinephrine support and of hospital stay in patients who received levosimendan.

**Table 3:** Outcome variables after inverse probability of treatment weighting

Variable	Levosimendan	Milrinone	Average treatment effect 95% CI	*P* value
Requiring epinephrine (proportions)	0.42	0.26	0.16 [− 0.03 to 0.34]	0.10
Epinephrine support (days)	1.00	1.82	− 0.82 [− 3.35 to 0.95]	0.25
VIS on postoperative day 1	8.60	13.86	− 5.26 [− 12.70 to − 1.36]	*0.02*
VIS on postoperative day 2	4.46	10.11	− 5.65 [− 13.68 to − 0.42]	*< 0.001*
VIS on postoperative day 3	2.57	6.84	− 4.28 [− 11.76 to − 1.13]	*< 0.001*
VIS on postoperative day 4	1.38	5.63	− 4.25 [− 11.89 to − 1.08]	*< 0.001*
Mechanical ventilation (h)	19.38	56.97	− 37.59 [− 138.85 to − 19.13]	*0.01*
CPIS within 48 h of admission	1.64	1.43	0.21 [− 0.41 to 0.81]	0.50
Renal replacement therapy (proportions)	0.03	0.08	− 0.05 [− 0.20 to 0.04]	0.26
Intensive care unit stay (days)	4.79	7.90	− 3.11 − 10.03 to − 1.48]	*0.009*
Hospital stay (days)	13.84	17.65	− 3.81 [− 15.58 to 3.88]	0.26

The 95% CI of the average treatment effect was estimated using bootstrapping with 500 re-sampling. Statistically significant results are shown in italics

*CPIS* Clinical Pulmonary Infection Score, *VIS* vasoactive inotropic score

## Discussion

We report the results of a retrospective study of outcomes following GUCH surgeries, where the patients received either levosimendan or milrinone postoperatively. Although the patients in the Levosimendan group had the highest risk scores, had more often preoperative ventricular dysfunction, and had longer surgeries, after adjustment by a propensity score including baseline and intraoperative variables, they had shorter durations of mechanical ventilation and ICU stay. This result should prompt prospective research to validate the use of levosimendan in the setting of GUCH surgery.

To date, this is the first study which compares levosimendan and milrinone during the perioperative care of GUCH surgeries. A randomized clinical trial of levosimendan vs milrinone in children less than 5 years of age undergoing congenital cardiac surgery has shown equivalence in terms of hemodynamic and biochemical parameters [9]. In neonates undergoing complex cardiac surgeries, a trial of levosimendan vs a more conventional inotropic strategy resulted in lower lactate levels [10]. A meta-analysis of levosimendan trials in paediatric cardiac surgery suggested that the drug was safe and provided a potential clinical benefit when applied to postoperative LCOS [8].

Data published in patients with acquired cardiac disease surgeries are conflicting. Both the LICORN and LEVO-CTS trials [14, 15] showed that a prophylactic 0.1 mcg kg^−1^ min^−1^ levosimendan infusion in patients with reduced LV ejection fraction did not result in a significantly lower rate of the short-term composite outcome (defined by a combination of LCOS criteria, requirement for mechanical ventricular assist device, renal replacement therapy, or death) when compared with placebo. The CHEETAH trial [16] showed no reduction in 30-day mortality when a very low 0.07 mcg kg^−1^ min^−1^ levosimendan dose was administered to patients with postoperative LCOS. These results are, however, subject of controversy [17]. The low levosimendan regimen in both the LICORN and the CHEETAH trials [14, 16] has been questioned, and details about hemodynamic monitoring are missing. In the CHEETAH trial [16], the patients started on levosimendan were already receiving high doses of epinephrine or dobutamine, which could have reduced the inotropic effect of levosimendan. The medical community agrees that some encouraging evidence of efficacy emerged from LEVO-CTS nevertheless [17]: the lower incidence of LCOS, lesser need for inotropic support by catecholamines, and improvement in CI indicate that levosimendan exhibited efficacy. Moreover, a subgroup analysis of the LEVO-CTS trial demonstrated that levosimendan was associated with lower 90-day mortality and LCOS in patients undergoing isolated coronary artery bypass grafting [18]. A systematic review pooling data from 28 280 patients included in 177 trials of inotropic-vasoactive support used to treat LCOS in vasoplegic syndromes, sepsis and cardiac surgery suggested that levosimendan was the only drug associated with improvement in survival [19]. As a result, a panel of 27 European experts reached a consensus on the recommendations proposed for the prophylactic use of levosimendan in cardiac surgery [20].

GUCH patients have higher risk of postoperative mortality and morbidity when compared with acquired cardiac disease patients. According to the Nationwide Inpatient Sample database, including data from over 2 million patients, the postoperative mortality following GUCH surgeries is 2.6% versus 1.8% following coronary artery bypass grafting (4). When compared with acquired cardiac disease patients, GUCH patients are at higher risk of arrhythmia (51.6 vs 29.8%), sepsis (7.24 vs 4.61%), thromboembolic complications (3.9 vs 1.4%) and neurologic complications (2.6 vs 0.9%)^4^. Postoperative mortality rates reported in smaller studies vary between 0.7% and 2% [12, 21–23], according to whether univentricular heart patients were included or not. The main risk factors of death include a NYHA category ≥ III, an altered RV function, emergency surgery [21], as well as pulmonary hypertension [23]. No patient in the present cohort died within 30 days of surgery, however long-term outcomes were not analysed. Two major risk factors of death, the NYHA category and an altered RV function, were found different between groups and were used to model the propensity score and to adjust for the probability of treatment assignment. Importantly, here we studied a very high risk GUCH population: in the 830 GUCH patients studied by Putman et al. over 17 years (5), less than 40% of the patients had NYHA III or IV category, whereas 60% of the patients in the present cohort were allocated to NYHA III or IV category.

The pathophysiological mechanism of cardiac failure in GUCH patients is complex^11^, associating: (i) volume overload through residual shunts and valvular regurgitations; (ii) pressure overload through intraventricular or valvular outflow tract obstructions; (iii) pulmonary hypertension; (iv) chronic cyanosis; (v) myocardial injury; (vi) arrhythmia; (vii) and inability of univentricular hearts (especially when following palliation of the hypoplastic left heart syndrome) to cope with the metabolic demand over the long term [24]. RV failure is very common in tetralogy of Fallot patients having undergone trans-annular patch repair, and who suffer of chronic pulmonary regurgitation [25], as well as in patients with chronic pulmonary hypertension subsequent to long-term left-to-right shunting [26, 27]. Additionally, pulmonary vascular resistances may increase postoperatively due to hypoxia, hypercapnia, acidosis, hypothermia, positive pressure ventilation and alpha-receptor stimulation. All together, these may explain the differences reported in the postoperative course of GUCH surgeries, when compared with acquired cardiac diseases. Here, 73.1% of all patients required inotropic-vasoactive support (49.3% of the patients received one inotropic-vasoactive agent, and 23.8% received two), which suggests that there was a high risk of LCOS in the present population (Fig. 2).

In 2016, the Working Group of Grown-Up Congenital Heart Disease and the Heart Failure Association of the European Society of Cardiology published the guidelines for the treatment of heart failure in GUCH patients^11^. Unfortunately, it did not provide guidelines for the management of acute heart failure, such as seen postoperatively. Since the use of catecholamines or phosphodiesterase-inhibitors in adults with cardiogenic shock is associated with increased mortality [28, 29], the use of newer drugs such as levosimendan to improve systolic function putatively without elevating intracellular calcium and without increasing the myocardial oxygen consumption [30] is appealing.

Milrinone, a conventional heart failure treatment in children and adults, selectively inhibits intracellular cardiac phosphodiesterase type 3, and the positive inotropic effect results through increased intracellular calcium levels. Its positive lusitropic properties, as well as systemic and pulmonary vasodilatation, make milrinone particularly useful when LCOS results from diastolic ventricular dysfunction and RV failure, or in case of pulmonary hypertension, such as often seen in GUCH patients. However, a significant increase in postoperative tachyarrhythmias [31] and mortality risk [32] has been reported.

Levosimendan binds cardiac troponin C and stabilizes calcium-induced conformational changes, which, in turn, promotes the prolonged interaction between actin and myosin filaments during systole. The resulting increase in contractility is unmatched by either milrinone or dobutamine [33, 34]. Levosimendan improves myocardial efficiency without an increase in the myocardial oxygen consumption [34], which reduces the risk of arrhythmia. Its vasodilatory effect is mediated by opening ATP-sensitive potassium channels in systemic, pulmonary, and coronary vascular smooth muscle cells. It has also been suggested that there was a potential for improvement of diastolic function [34].

Levosimendan has been shown to provide hemodynamic support in a wide range of critical illness situations, including cardiogenic or septic shock, weaning from mechanical ventilation, weaning from extracorporeal membrane oxygenation and cardiorenal syndrome [35]. Levosimendan has a positive impact on several specific conditions related to GUCH surgeries. As aforementioned, GUCH patients often present with a failing RV, and a recent meta-analysis reported improved RV function when levosimendan was used to treat RV failure in a variety of heart and lung diseases [36]. Levosimendan induces vasodilatation in the pre-constricted pulmonary circulation [37], which is of particular relevance in the post-CPB setting. By improving the diastolic function, it is of particular interest when LCOS is due to LV or RV diastolic dysfunction, a common finding in patients with residual obstruction of the left or right outflow tract. Experimental work demonstrated an increase in the RV myocardial efficiency when levosimendan was used to treat RV hypertrophy and failure [38], and it has been proposed for the treatment of RV failure in patients with pulmonary hypertension [39].

Vasopressors are unsuitable for GUCH patients, they have a negative impact on the myocardium [40] and on the microcirculation [41], and their use has been linked with poor outcomes after GUCH surgeries [29]. Therefore, the choice made by the authors was to privilege levosimendan, milrinone and volume load, and to associate low dosages of epinephrine and/or norepinephrine in case of systemic hypotension and evidence of inappropriate organ perfusion. Both vasopressors were weaned as soon as possible. Importantly, patients in the Levosimendan group were weaned from epinephrine earlier than patients in the Milrinone group (Table 3), and had lower VIS during the early postoperative days.

The main finding here was a shorter duration of mechanical ventilation and ICU stay in the Levosimendan group. Importantly, the CPIS score was similar in the Levosimendan and in the Milrinone groups, showing that the difference in ventilation durations was not due to the occurrence of postoperative pneumopathy. We could not infer any hypothesis about the pathophysiological pathways leading to this beneficial effect based on this retrospective data analysis. Literature contains several reports linking the use of levosimendan to successful weaning from mechanical ventilation, either due to recovery of myocardial dysfunction [42, 43] or to recovery of diaphragmatic dysfunction [44–46]. We hypothesize that the beneficial effect is partly due to its pharmacological properties, since the concentration of the long-lasting active metabolite OR 1896 remains stable up to 8 days after a 24 h infusion, and enables to overcome the increase in the metabolic demand during ventilation weaning through a residual positive inotropic effect.

### Limitations

The results of this single-centre and small-sampled retrospective study need to be interpreted with caution, and require validation in prospective trials. Because of the huge initial unbalance between groups, the patients in the Control group were not included in the outcome analysis. Due to missing data inherent to the retrospective design of the study, several hemodynamic and echocardiographic parameters could not be analysed, therefore it was not possible to infer hypothesis about the pathophysiological pathways leading to the beneficial effect of levosimendan. The use of the VIS might have favoured the Levosimendan group here, since the VIS does not account for the vasoactive and inotropic effect of levosimendan. Due to missing data, long-term outcomes were not analysed either.

## Conclusion

Critical care management requires an in-depth understanding of underlying pathophysiology of GUCH patients in order to apply contemporary concepts of adult intensive care to this specific population. Our study suggests that when used following surgery in high-risk GUCH patients, levosimendan has a beneficial impact on the duration of mechanical ventilation, of ICU stay, and on inotropic requirements. Further research is needed to validate the use of levosimendan in this setting.

## Data Availability

The datasets used and/or analysed during the current study are available from the corresponding author on reasonable request.
